# Supplementary material for: Jointly Inferring the Dynamics of Population Size and Sampling Intensity from Molecular Sequences
Source: Mol Biol Evol. 2020 Jan 31;37(8):2414–29. doi: 10.1093/molbev/msaa016 (PMC7403618; doi:10.1093/molbev/msaa016)
Supplement: msaa016_Supplementary_Data [file msaa016_supplementary_data.zip › msaa016-suppl-data/pref_supplement.pdf]

Supplementary Material

Jointly inferring the dynamics of population size and sampling intensity from molecular sequences

KV Parag[1,2,†, *], L du Plessis[1,†, *], and OG Pybus[1, *]

[1]Department of Zoology, University of Oxford, Oxford, OX1 3SY, UK
[2]MRC Centre for Global Infectious Disease Analysis, Imperial College London, London, W2 1PG, UK
[†]Equal contribution
[*]**E-mail:** k.parag@imperial.ac.uk, louis.duplessis@zoo.ox.ac.uk, oliver.pybus@zoo.ox.ac.uk

November 25, 2019

Contents

A Supplementary Methods **2**
- A.1 Bayesian Implementation Simulation Study . . . 2
  - A.1.1 Simulations and inferences . . . 2
  - A.1.2 Summary statistics . . . 3
- A.2 Case Study 1: Seasonal Human Influenza . . . 3
- A.3 Case Study 2: Steppe Bison . . . 4
- A.4 References . . . 6

B Supplementary Figures **6**

A Supplementary Methods

A.1 Bayesian Implementation Simulation Study

A.1.1 Simulations and inferences

We used the `phylodyn` R package (Karcher et al., 2017) to simulate heterochronously sampled coalescent trees under five population size trajectories, $N(t)$, given in table S1. Trees were simulated with approximately 500 samples, sampled between $t = 0$ and $t := \min\{48, \lfloor t_{10} \rfloor\}$ where $t_{10} = \{t : N(t) < 10\}$. The sampling period was divided into 24 equally-spaced epochs, with the sampling intensity (β) during each epoch set such that an approximately equal number of samples is drawn from each epoch. We used this procedure to simulate 100 replicate trees for each population size trajectory.

Table S1: Population size trajectories used in the Bayesian implementation simulation study.

Constant size	$N(t) = 100$	
Bottleneck	$N(t) = \begin{cases} 100 \\ 10 \end{cases}$	$t \leq 10,\ t \geq 20$ $10 < t < 20$
Boom-bust	$N(t) = \begin{cases} 500\exp(t-2) \\ 500\exp(2-t) \end{cases}$	$t \leq 2$ $t > 2$
Cyclical boom-bust	$N(t) = \begin{cases} 200\exp\Big(-5\big(t \pmod{10}\big)\Big) \\ 200\exp\Big(5\big[\big(t \pmod{10}\big) - 10\big]\Big) \end{cases}$	$t \pmod{10} \leq 5$ $t \pmod{10} > 5$
Logistic growth and decline	$N(t) = \begin{cases} 10 + \frac{190}{1+\exp\Big(2\big[3-\big(t \pmod{12}\big)\big]\Big)} \\ 10 + \frac{190}{1+\exp\Big(2\big[3+\big(t \pmod{12}\big)-12\big]\Big)} \end{cases}$	$t \pmod{12} \leq 6$ $t \pmod{12} > 6$

Inferences were performed for each simulated tree using the BESP implementation in BEAST2 v2.6 (Bouckaert et al., 2019). To estimate N we set $p = 100$, and grouped equal numbers of events (coalescent/sampling) so that each population size segment had roughly 10 informative events i.e. $k_j \approx 10$ for all j. To estimate β we set $p' = 24$, with the boundaries of sampling epochs chosen to coincide with the time of the first sampling event after each sampling epoch change (going into the past). Because each simulation contains $\approx$500 samples, in practice this resulted in sampling intensity change-point times that were very close to the true epoch times used in the simulations. In all analyses N and β were jointly estimated, and all other parameters (including the tree) were fixed to the truth.

MCMC chains were run for 20 million iterations and parameters sampled every 20,000 iterations. Convergence was checked by calculating the effective sample size (ESS) of parameters using the `coda` R pack-

age (Plummer et al., 2006) after discarding 10% of samples as burn-in and confirming that all parameters had ESS values greater than 200. Workflows for the Bayesian simulation study are availabe at `https://github.com/laduplessis/BESP_paper-analyses`.

A.1.2 Summary statistics

Let θ_i be the true values of a vector of parameters, where θ_i is defined over the period $\left[t_{i-1}, t_i\right)$, with $t_i > t_{i-1}$ for $1 \leq i \leq p$. If $\hat{\theta}_i$ is the median *a posteriori* estimate of θ_i and $\left[\hat{\theta}_{i,L}, \hat{\theta}_{i,U}\right]$ is the HPD interval we calculate the following statistics:

i. *Mean relative bias:*

$$\frac{1}{t_p - t_0} \sum_{i=1}^{p} \frac{\left(\hat{\theta}_i - \theta_i\right)}{\theta_i} \left(t_i - t_{i-1}\right)$$

ii. *Mean relative HPD interval width:*

$$\frac{1}{t_p - t_0} \sum_{i=1}^{p} \frac{\left|\hat{\theta}_{i,U} - \hat{\theta}_{i,L}\right|}{\theta_i} \left(t_i - t_{i-1}\right)$$

iii. *Mean coverage:*

$$\frac{1}{t_p - t_0} \sum_{i=1}^{p} 1_{[\hat{\theta}_{i,U}, \hat{\theta}_{i,L}]}(\theta_i) \left(t_i - t_{i-1}\right)$$

We use the above three statistics to calculate summary statistics for N (across population size segments) and β (across sampling epochs), between the most recent and most ancient samples. Since the true population size trajectory, $N(t)$ varies across each segment we use the harmonic mean of $N(t)$ within a segment as the true parameter value. This is a lower bound on the true parameter estimated by the BESP, with the true estimated parameter being a product of the harmonic and arithmetic means (see equation 2 and Materials and Methods in the main text). By the simulation design β does not vary substantially over each sampling epoch and we simply use the true β to compute its summary statistics.

A.2 Case Study 1: Seasonal Human Influenza

We analysed a subset of the 687 human influenza A/H3N2 HA gene sequences sampled in New York state between 1993 and 2005 that were used in the analyses presented in Rambaut et al. (2008). We removed all sequences sampled during the 1992/1993 influenza season, as only isolates from the second half of this season were included in the dataset. The remaining dataset contains 637 sequences representing 12 complete influenza seasons (1993/1994–2004/2005). The HA alignment is the same as that in Rambaut et al. (2008) but with 50 sequences removed and comprises the coding regions of the HA segment and spans 1,698 bp.

We used an SRD06 substitution model (Shapiro et al., 2006), that allows for different rates of evolution of the first and second codon positions relative to the third, and uses an HKY nucleotide substitution model with Γ-distributed rate heterogeneity for both partitions. We further utilised an uncorrelated lognormal relaxed clock model (Drummond et al., 2006) to allow for variations in the overall rate of molecular evolution across branches in the tree. Default priors were used for the nucleotide substitution model and an informative lognormal prior was placed on the mean molecular clock rate, with a mean of $6 \times 10^{-}3$ s/s/y and $S = 0.1$.

Table S2: Sampling epochs used for the 12-epoch BESP on the influenza A/H3N2 dataset.

Season	Samples	Start	End	Years
2004/2005	80	2005.25	2004.73	0.52
2003/2004	85	2004.73	2003.64	1.09
2002/2003	16	2003.64	2003.07	0.57
2001/2002	84	2003.07	2001.91	1.16
2000/2001	1	2001.91	2001.08	0.83
1999/2000	70	2001.08	1999.76	1.32
1998/1999	78	1999.76	1998.85	0.91
1997/1998	49	1998.85	1997.92	0.93
1996/1997	51	1997.92	1996.88	1.04
1995/1996	25	1996.88	1995.92	0.96
1994/1995	49	1995.92	1994.59	1.33
1993/1994	49	1994.59	1993.87	0.72

For analyses with the BESP a uniform prior with an upper bound of 1×10^6 was placed on β. As a tree-prior we used either the BESP or BSP, with three different configurations:

i. **BESP (12 epochs):** $p = 40,\ p' = 12,\ k_j \geq 2,\ t_j - t_{j-1} > 0.08$ years ($\approx$ 1 month) for population size segments and sampling epochs. Sampling epoch group sizes (K') and change-point times are fixed as in table S2.

ii. **BESP (1 epoch):** $p = 40,\ p' = 1,\ k_j \geq 2,\ t_j - t_{j-1} > 0.08$ years ($\approx$ 1 month) for population size segments. β is constant between 2005.25 and 1993.87 and 0 between 1993.87 and the time of the most recent common ancestor.

iii. **BSP:** $p = 40,\ k_j \geq 2,\ t_j - t_{j-1} > 0.08$ years ($\approx$ 1 month) for population size segments.

To aid convergence we computed an initial maximum-likelihood genetic distance tree using RAxML v8 (Stamatakis, 2014) under a general time reversible nucleotide substitution model with Γ-distributed rate heterogeneity. We then used TreeTime (Sagulenko et al., 2018) to construct a time-calibrated initial tree with branch lengths in years. The models we use differ from the model used in Rambaut et al. (2008) only in the prior distributions, initial tree and bounds on the number of events in each segment/epoch and segment/epoch lengths. Rambaut et al. (2008) only used the BSP (with no minimum group size), set no explicit priors for clock and substitution model parameters and imposed no bounds on segment parameters.

All analyses were performed in BEAST v2.6 (Bouckaert et al., 2019). For each model, we computed 7 independent MCMC chains of 200 million iterations and sampled parameters and trees every 10,000 iterations. We used custom R scripts to combine chains after discarding 30% of samples as burn-in. The combined chain was thinned by a factor of 3 and convergence was checked by calculating the ESS of parameters using the `coda` R package (Plummer et al., 2006). The marginal posterior estimates of $N(t)$ were obtained by discretising the N_j parameters over an even grid of 80 cells, between 2005.25 and 1992.25 using a custom R script. To compute the maximum clade credibility (MCC) tree, we used the program `logcombiner` to combine the 7 sets of tree samples after discarding 30% of samples and thinning by a factor of 3. The program `treeannotator` was then used to compute the MCC tree of the resulting posterior tree distribution. Workflows for the seasonal human influenza case study are availabe at `https://github.com/laduplessis/BESP_paper-analyses`.

A.3 Case Study 2: Steppe Bison

We analysed 152 bison mtDNA control region sequences of 602 bp, dating from the present to 55,182 years BP. The alignment is the same as the one used in Gill et al. (2012). In the analyses we used an HKY+Γ

Table S3: Specification of the sampling epochs for the bison dataset.

Epoch	Samples	Start (BP)	End (BP)	Years
1	24	0	450	450
2	16	450	3,903	3,453
3	13	3,903	9,068	5,165
4	19	9,068	14,753	5,685
5	9	14,753	19,815	5,062
6	9	19,815	24,752	4,937
7	9	24,752	29,377	4,625
8	12	29,377	34,987	5,610
9	16	34,987	39,836	4,849
10	12	39,836	44,598	4,762
11	5	44,598	49,975	5,377
12	8	49,975	55,182	5,207

substitution model (Hasegawa et al., 1985; Yang, 1994) and a strict clock model. Default priors were used for the nucleotide substitution model and a uniform prior with an upper bound of 0.1 s/s/y was placed on the molecular clock rate. For analyses with the BESP a uniform prior with an upper bound of 1×10^6 was placed on β. All analyses were started from a random constant population size coalescent tree. As a tree-prior we used either the BESP or BSP, with three different configurations:

i. **BESP (12 epochs):** $p = 20,\ p' = 12,\ k_j \geq 2,\ t_j - t_{j-1} > 100$ years for population size segments and sampling epochs. Sampling epoch group sizes (K') and change-point times are fixed as in table S3.

ii. **BESP (1 epoch):** $p = 20,\ p' = 1,\ k_j \geq 2,\ t_j - t_{j-1} > 100$ years for population size segments. β is constant between the present and 55,182 years BP and 0 between 55,182 years BP and the time of the most recent common ancestor.

iii. **BSP:** $p = 20,\ k_j \geq 2,\ t_j - t_{j-1} > 100$ years for population size segments.

All analyses were performed in BEAST v2.6 (Bouckaert et al., 2019). For each model, we computed 3 independent MCMC chains of 200 million iterations and sampled parameters and trees every 10,000 iterations. We used a custom R script to combine chains after discarding 10% of samples as burn-in. The combined chain was thinned by a factor of 3 and convergence was checked by calculating the ESS of parameters using the `coda` R package (Plummer et al., 2006). The marginal posterior estimates of $N(t)$ were obtained by discretising the N_j parameters over an even grid of 200 cells, between the present and 80 ka BP using a custom R script. Workflows for the steppe bison case study are availabe at `https://github.com/laduplessis/BESP_paper-analyses`.

A.4 References

Bouckaert, R et al. (2019). “BEAST 2.5: An Advanced Software Platform for Bayesian Evolutionary Analysis”. In: *PLoS Comp. Biol* 15.4, e1006650.

Drummond, Alexei J. et al. (2006). “Relaxed Phylogenetics and Dating with Confidence”. In: *PLOS Biology* 4.5, e88.

Gill, M et al. (2012). “Improving Bayesian Population Dynamics Inference: A Coalescent-Based Model for Multiple Loci”. In: *Mol. Biol. Evol* 30.3, pp. 713–24.

Hasegawa, Masami et al. (1985). “Dating of the human-ape splitting by a molecular clock of mitochondrial DNA”. In: *Journal of Molecular Evolution* 22.2, pp. 160–174.

Karcher, M et al. (2017). “PHYLODYN: an R package for Phylodynamic Simulation and Inference”. In: *Mol. Ecol. Res* 17, pp. 96–100.

Plummer, Martyn et al. (2006). “CODA: Convergence Diagnosis and Output Analysis for MCMC”. In: *R News* 6.1, pp. 7–11. URL: `https://journal.r-project.org/archive/`.

Rambaut, A et al. (2008). “The Genomic and Epidemiological Dynamics of Human Influenza A Virus”. In: *Nature* 453.7195, pp. 615–619.

Sagulenko, Pavel et al. (2018). “TreeTime: Maximum-likelihood phylodynamic analysis”. en. In: *Virus Evolution* 4.1.

Shapiro, Beth et al. (2006). “Choosing Appropriate Substitution Models for the Phylogenetic Analysis of Protein-Coding Sequences”. In: *Molecular Biology and Evolution* 23.1, pp. 7–9.

Stamatakis, Alexandros (2014). “RAxML version 8: a tool for phylogenetic analysis and post-analysis of large phylogenies”. en. In: *Bioinformatics* 30.9, pp. 1312–1313.

Yang, Ziheng (1994). “Maximum likelihood phylogenetic estimation from DNA sequences with variable rates over sites: Approximate methods”. In: *Journal of Molecular Evolution* 39.3, pp. 306–314.

B Supplementary Figures

S1 Constant population size trajectory simulation example 7
S2 Bottleneck population size trajectory simulation example 8
S3 Boom-bust population size trajectory simulation example 9
S4 Cyclical population size trajectory simulation example 10
S5 Logistic growth and decline population size trajectory simulation example 11
S6 Seasonal Human Influenza case study, 12-epoch BESP compared to single-epoch BESP 12
S7 Seasonal Human Influenza case study population size segments estimated under different models 13
S8 Seasonal Human Influenza case study MCC tree estimated under the 12-epoch BESP 14
S9 Steppe Bison case study, 12-epoch BESP compared to single-epoch BESP 15
S10 Steppe Bison case study population size segments estimated under different models 16
S11 Steppe Bison case study MCC tree estimated under the 12-epoch BESP 17

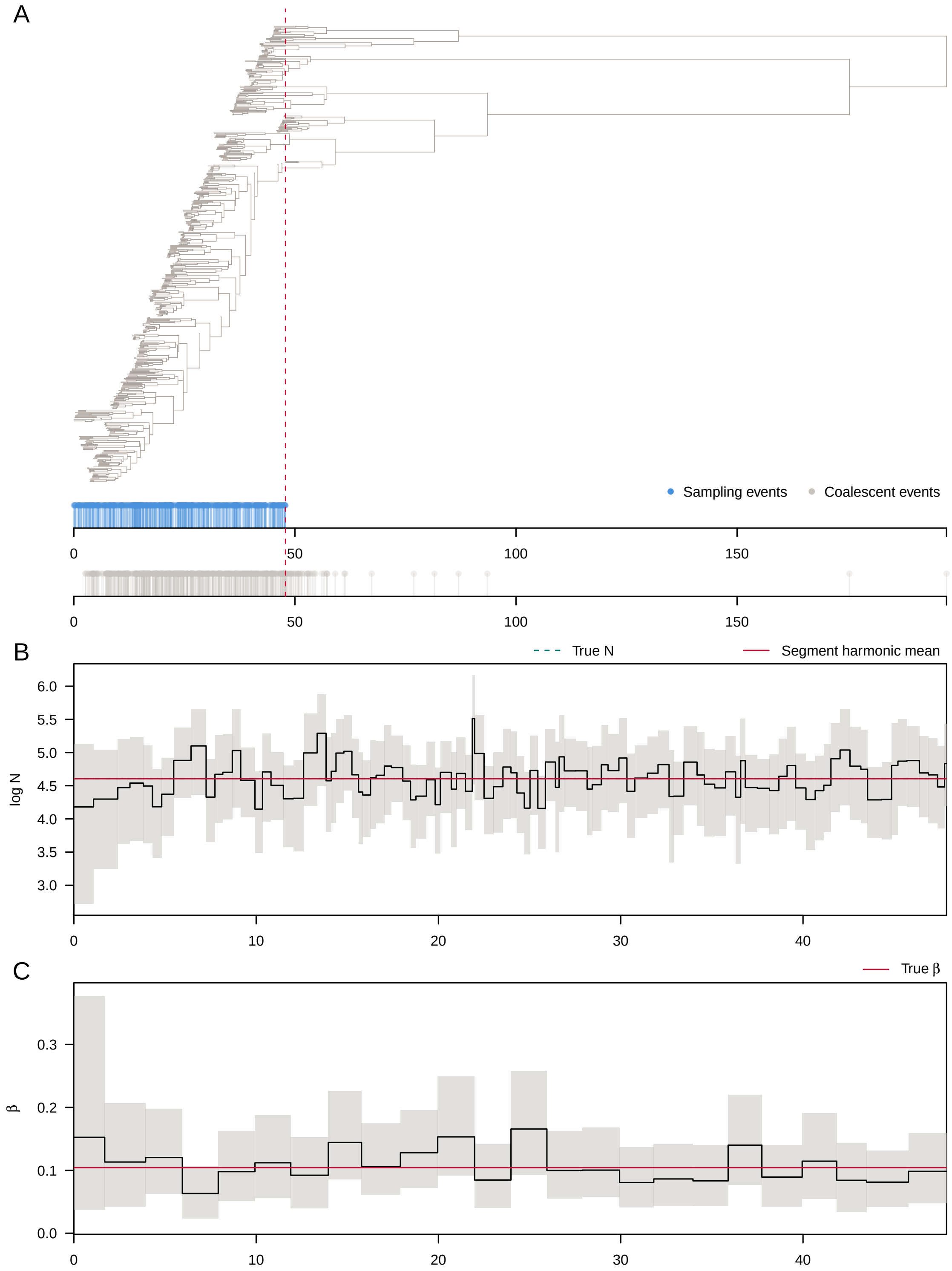

Figure S1: (A) Example of one of the 100 replicate trees simulated under the constant size demographic scenario. Sampling (blue) and coalescent (grey) events are shown below. The red dashed line indicates the time of the most ancient sample. (B) Median (solid black line) and HPD intervals (shaded areas) for the effective population size (N) estimates between the most recent and most ancient samples. The dashed green line shows the true N-trajectory used to simulate the tree in A and the red line the harmonic mean of the true N during each segment. (C) Median (solid black line) and HPD intervals (shaded areas) for the sampling intensity (β) estimates for each sampling epoch. The red line shows the true β used to simulate the tree in A.

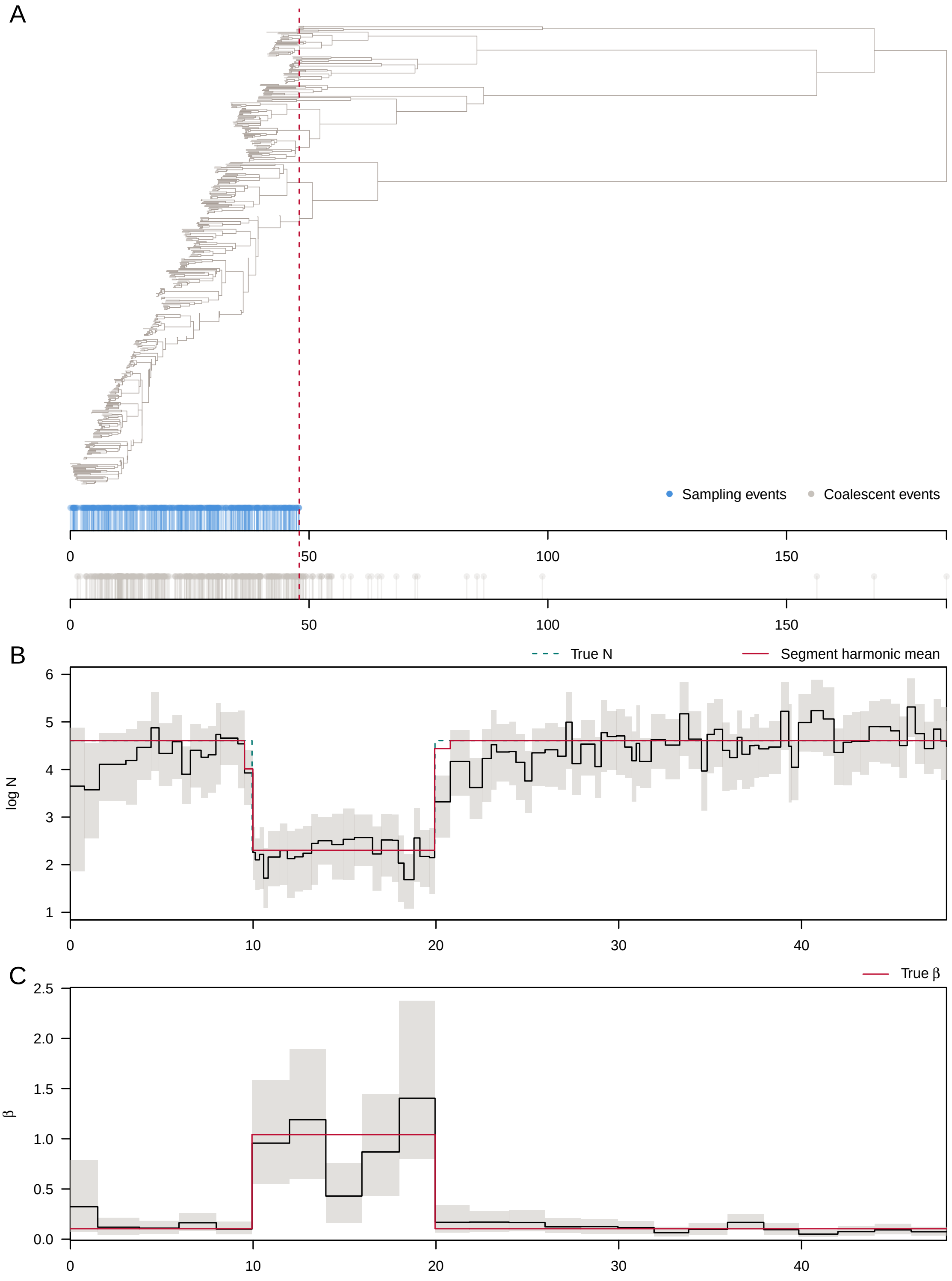

Figure S2: (A) Example of one of the 100 replicate trees simulated under the bottleneck demographic scenario. Sampling (blue) and coalescent (grey) events are shown below. The red dashed line indicates the time of the most ancient sample. (B) Median (solid black line) and HPD intervals (shaded areas) for the effective population size (N) estimates between the most recent and most ancient samples. The dashed green line shows the true N-trajectory used to simulate the tree in A and the red line the harmonic mean of the true N during each segment. (C) Median (solid black line) and HPD intervals (shaded areas) for the sampling intensity (β) estimates for each sampling epoch. The red line shows the true β used to simulate the tree in A.

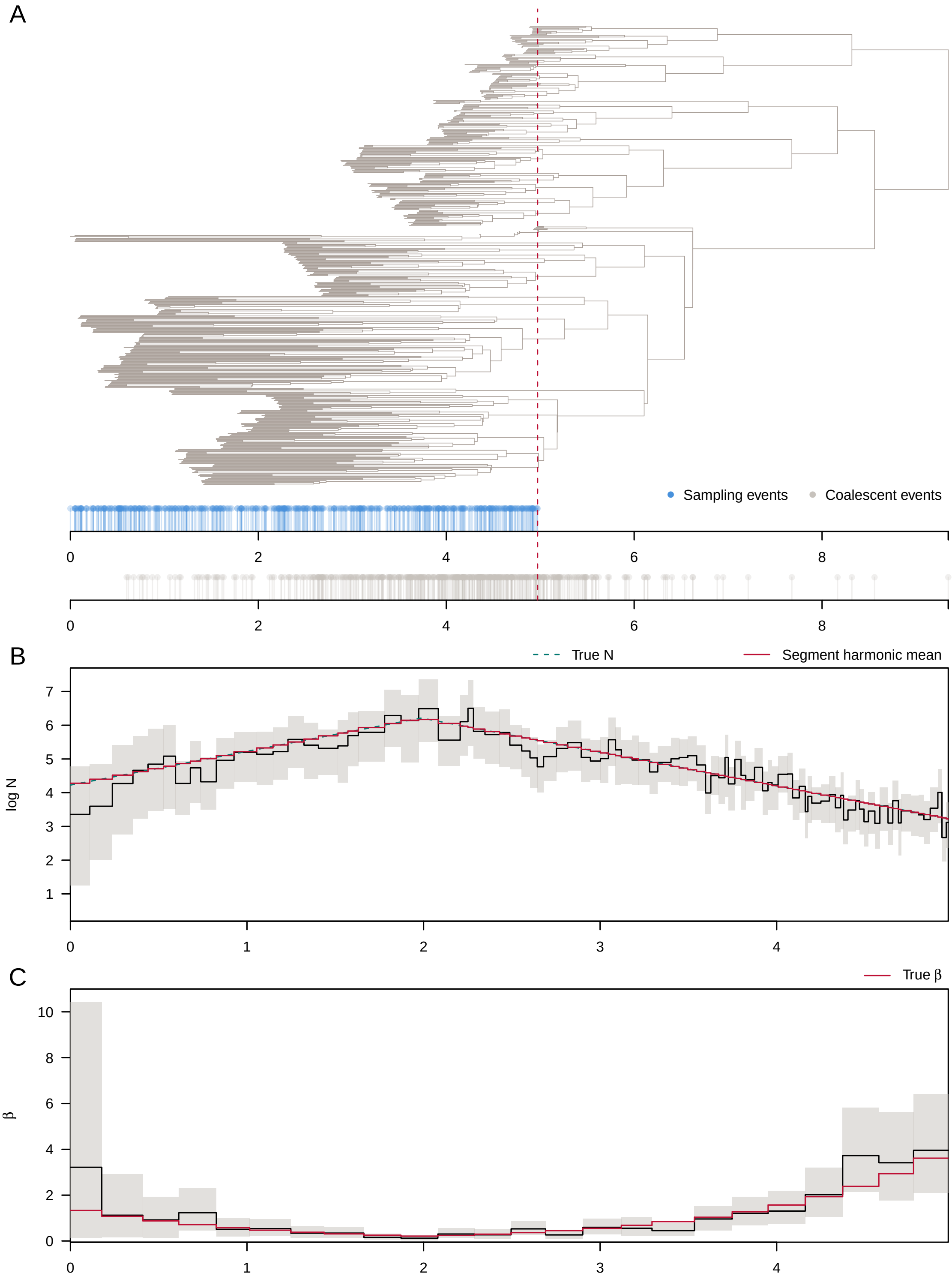

Figure S3: (A) Example of one of the 100 replicate trees simulated under the boom-bust demographic scenario. Sampling (blue) and coalescent (grey) events are shown below. The red dashed line indicates the time of the most ancient sample. (B) Median (solid black line) and HPD intervals (shaded areas) for the effective population size (N) estimates between the most recent and most ancient samples. The dashed green line shows the true N-trajectory used to simulate the tree in A and the red line the harmonic mean of the true N during each segment. (C) Median (solid black line) and HPD intervals (shaded areas) for the sampling intensity (β) estimates for each sampling epoch. The red line shows the true β used to simulate the tree in A.

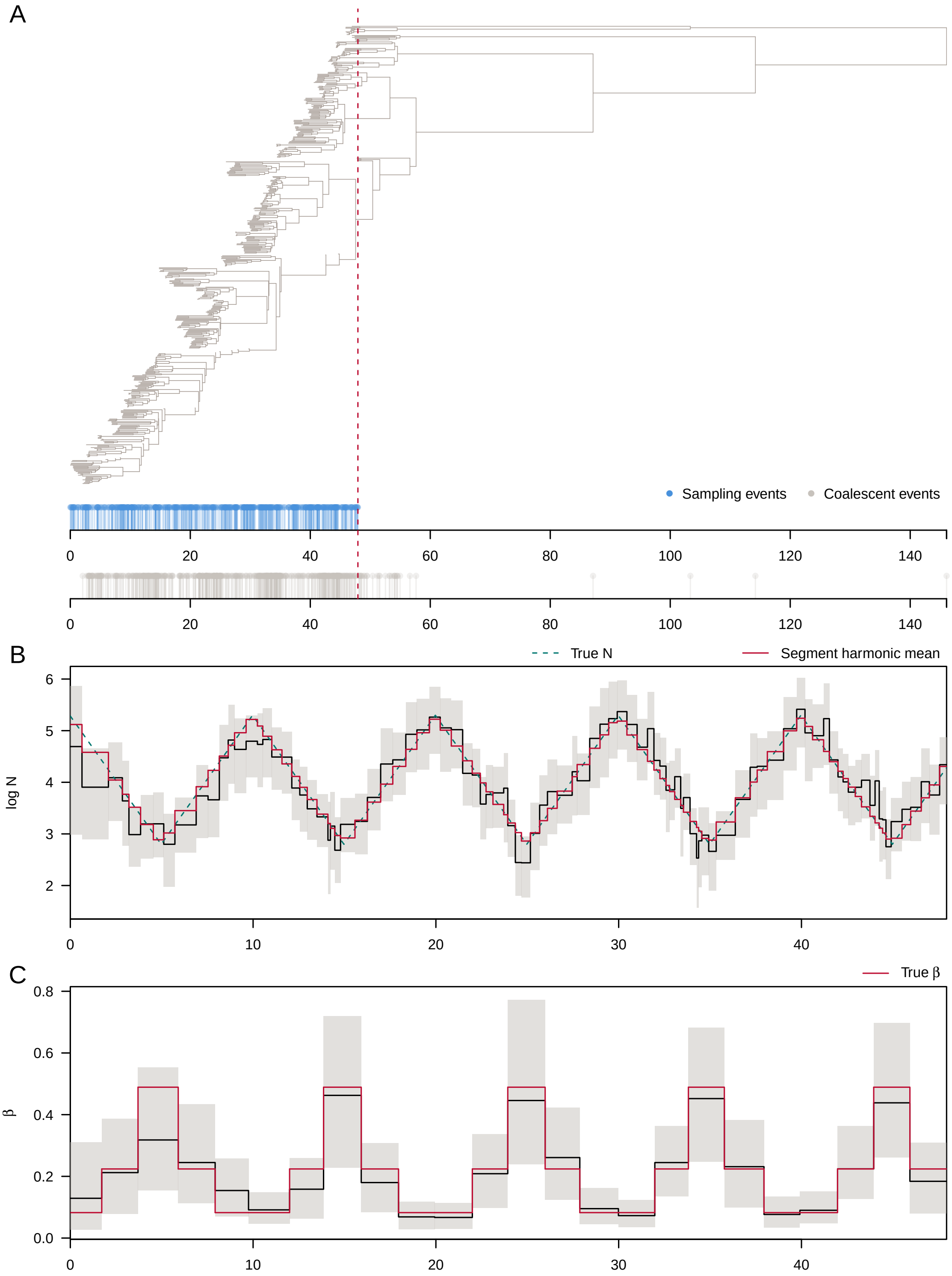

Figure S4: (A) Example of one of the 100 replicate trees simulated under the cyclical boom-bust demographic scenario. Sampling (blue) and coalescent (grey) events are shown below. The red dashed line indicates the time of the most ancient sample. (B) Median (solid black line) and HPD intervals (shaded areas) for the effective population size (N) estimates between the most recent and most ancient samples. The dashed green line shows the true N-trajectory used to simulate the tree in A and the red line the harmonic mean of the true N during each segment. (C) Median (solid black line) and HPD intervals (shaded areas) for the sampling intensity (β) estimates for each sampling epoch. The red line shows the true β used to simulate the tree in A.

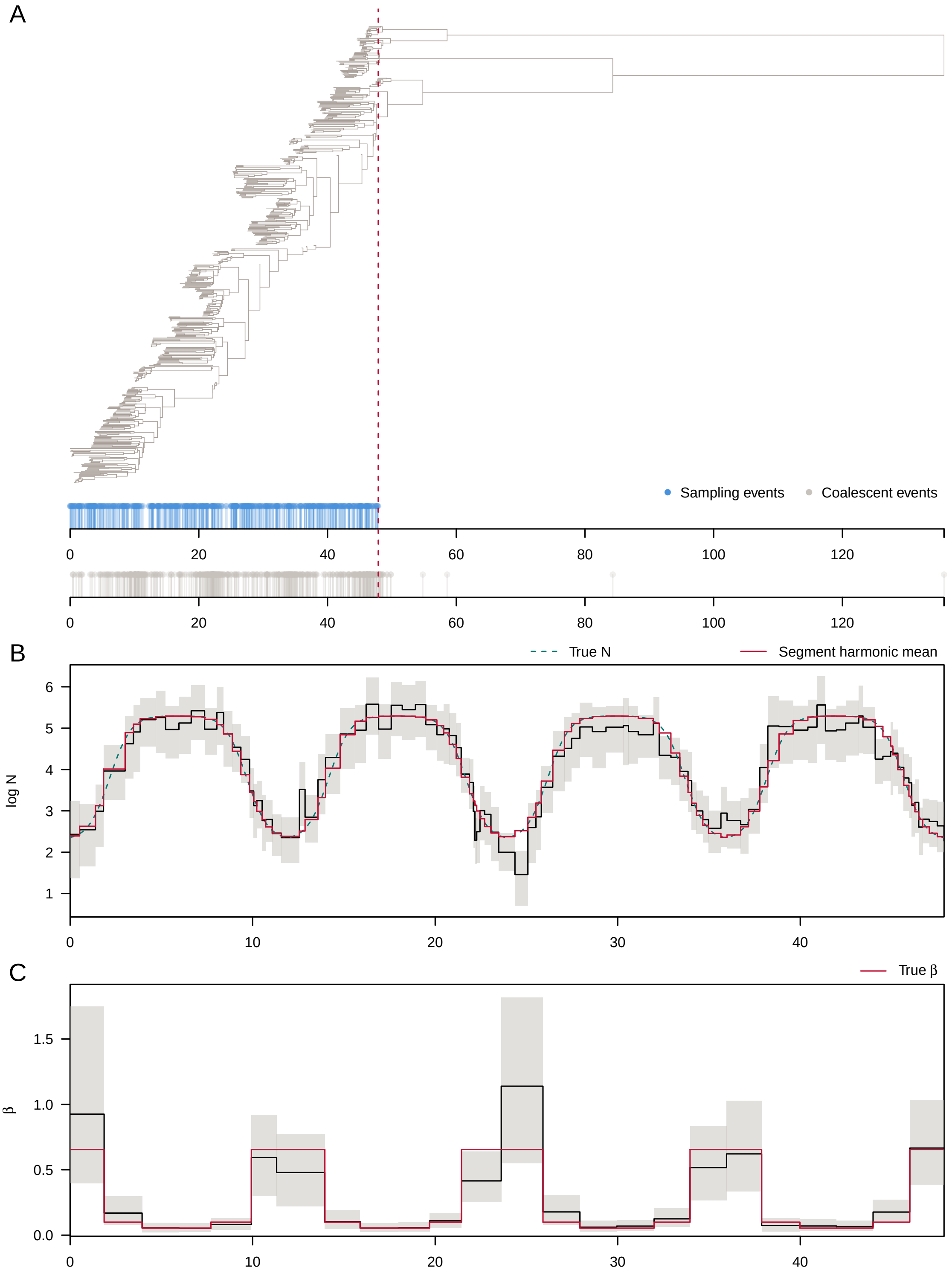

Figure S5: (A) Example of one of the 100 replicate trees simulated under the logistic growth and decline demographic scenario. Sampling (blue) and coalescent (grey) events are shown below. The red dashed line indicates the time of the most ancient sample. (B) Median (solid black line) and HPD intervals (shaded areas) for the effective population size (N) estimates between the most recent and most ancient samples. The dashed green line shows the true N-trajectory used to simulate the tree in A and the red line the harmonic mean of the true N during each segment. (C) Median (solid black line) and HPD intervals (shaded areas) for the sampling intensity (β) estimates for each sampling epoch. The red line shows the true β used to simulate the tree in A.

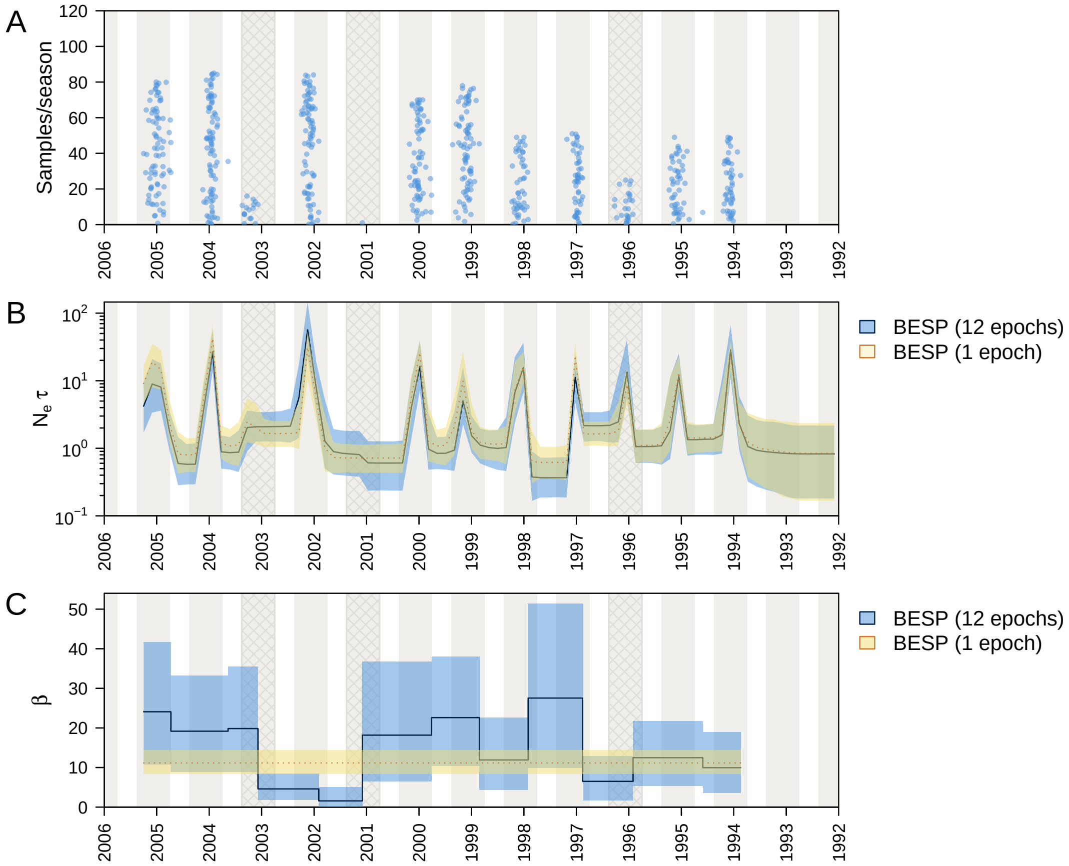

Figure S6: (A) Density of sequence sampling dates through time for the alignment of 637 A/H3N2 HA sequences from NY state that we analysed. Blue dots indicate stripcharts of individual samples for each season. The stripchart heights give the number of samples in each season. Grey shading indicates the approximate period of influenza observation in New York state during each season (epidemiological week 40, to week 20 in the next year). Cross-hatched seasons are those where A/H3N2 was not the dominant influenza virus subtype. (B) Median (solid/dotted line) and 95% highest posterior density (HPD) intervals (shaded areas) for the genetic diversity estimates ($N_e\tau$) through time. The 12-epoch BESP estimate is shown in blue and the single-epoch BESP estimate is in yellow. (C) Median (solid line/dotted line) and 95% HPD intervals (shaded areas) of the estimated sampling intensities (β) for each sampling epoch. The 12-epoch BESP estimates are shown in blue and a single-epoch (density-defined) estimate is in yellow.

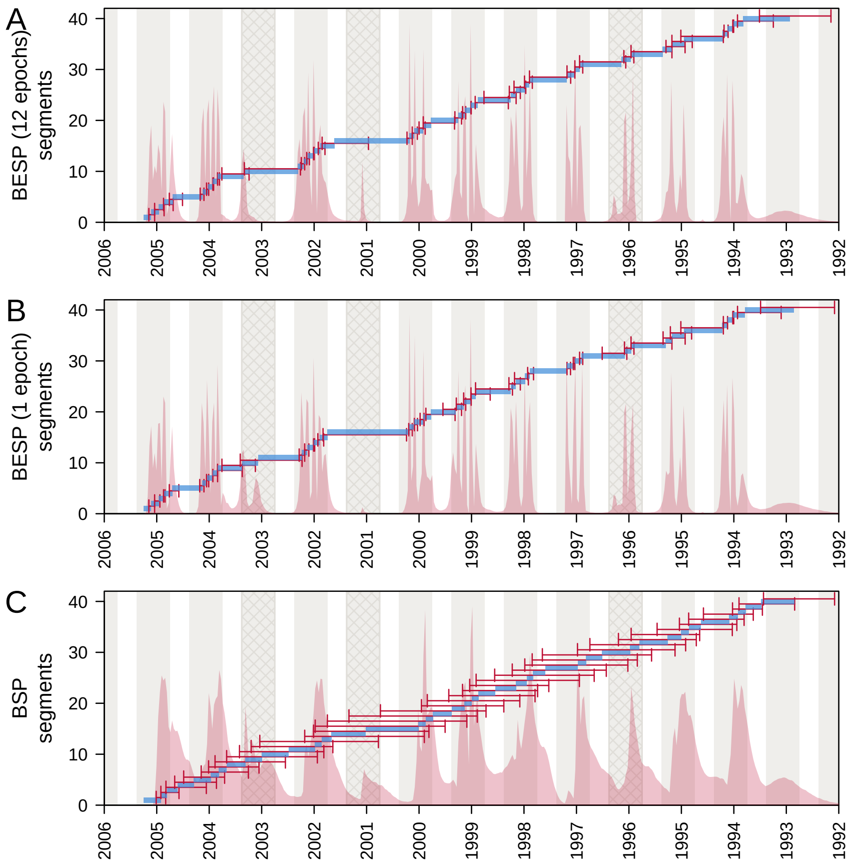

Figure S7: Population size segments for the alignment of 637 A/H3N2 HA sequences from NY state, as estimated under the 12-epoch BESP (A), single-epoch BESP (B) and BSP (C), with $p = 40$. Median posterior estimates of segments (t_{j-1}–t_j) are shown in blue. HPD intervals for the segment end-times are indicated by red arrows. Red shading shows the kernel density estimate of the posterior segment times (t_j). Grey shading indicates the approximate period of influenza observation in New York state during each season (epidemiological week 40, to week 20 in the next year). Cross-hatched seasons are those where A/H3N2 was not the dominant influenza virus subtype.

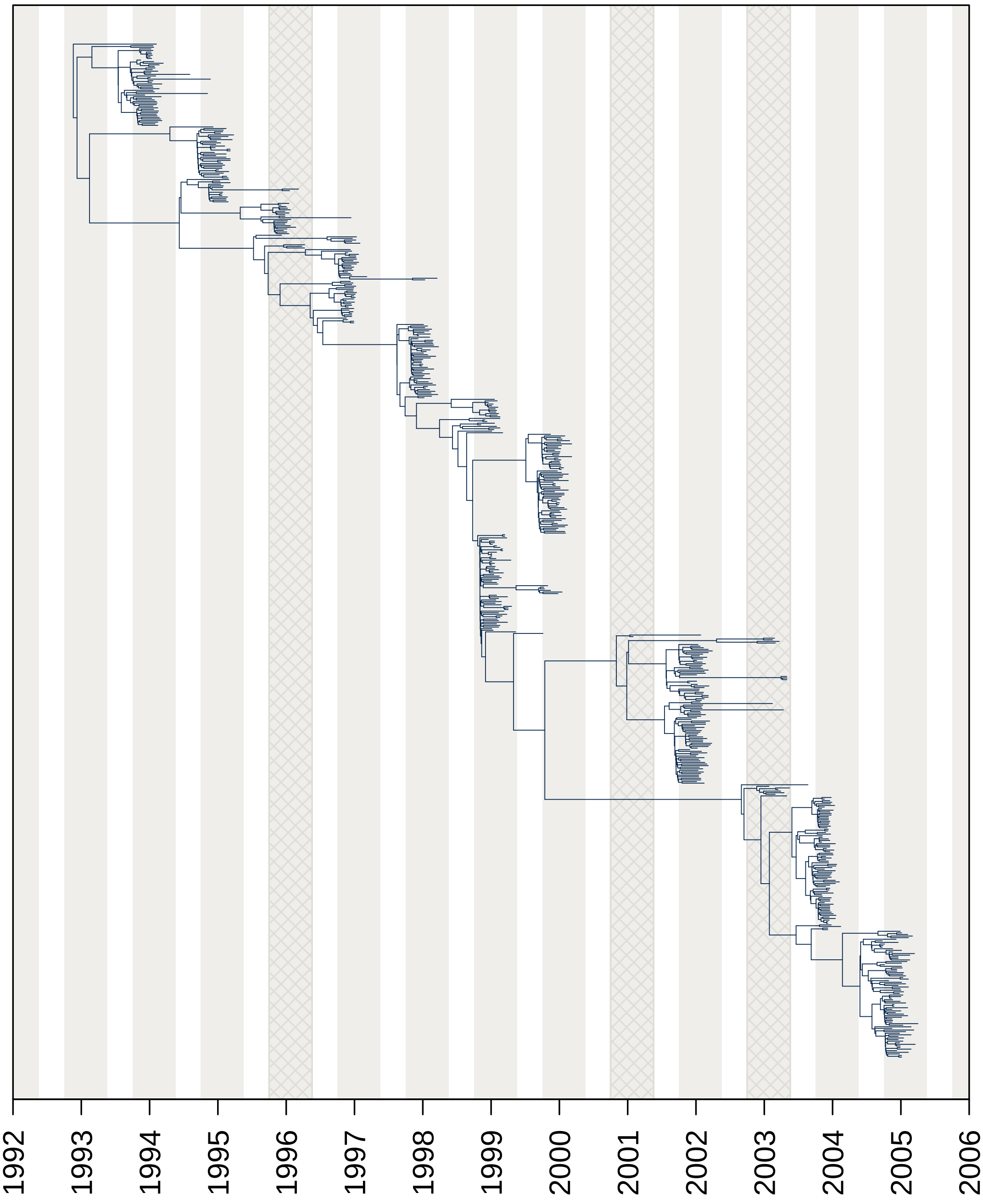

Figure S8: MCC tree of the alignment of 637 A/H3N2 HA sequences estimated under the 12-epoch BESP. Grey shading indicates the approximate period of influenza observation in New York state during each season (epidemiological week 40, to week 20 in the next year). Cross-hatched seasons are those where A/H3N2 was not the dominant influenza virus subtype.

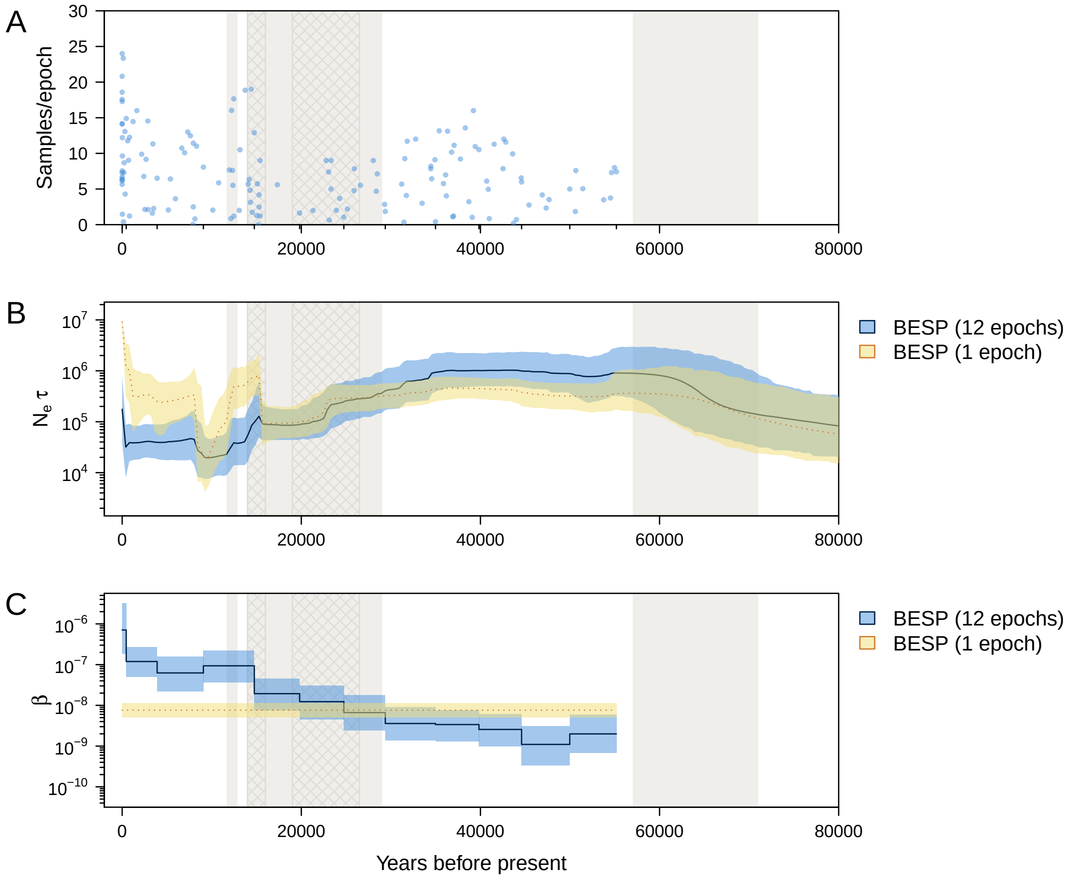

Figure S9: (A) Density of sequence sampling dates through time for the alignment of 152 bison mtDNA sequences that we used. Blue dots indicate stripcharts of individual samples for each sampling epoch. The height of the stripcharts is equal to the number of samples in each epoch. Small tick marks on the x-axis represent epoch times. Grey shading indicates cool periods in the Earth's climate (from the present: Younger Dryas, Marine Isotope Stages (MIS) 2, MIS 4). The two cross hatched areas delimit the time of the last glacial maximum (≈26.5–19 ka BP) and approximate time of substantial human settlement of the Americas (≈16–14 ka BP). (B) Median (solid/dotted line) and 95% highest posterior density (HPD) intervals (shaded areas) for the genetic diversity estimates ($N_e\tau$) through time. The 12-epoch BESP estimate is shown in blue and the single-epoch BESP estimate in yellow. (C) Median (solid line/dotted line) and 95% HPD intervals (shaded areas) of the estimated sampling intensities (β) for each sampling epoch. The 12-epoch BESP estimates are in blue and a single-epoch (density-defined) estimate is in yellow.

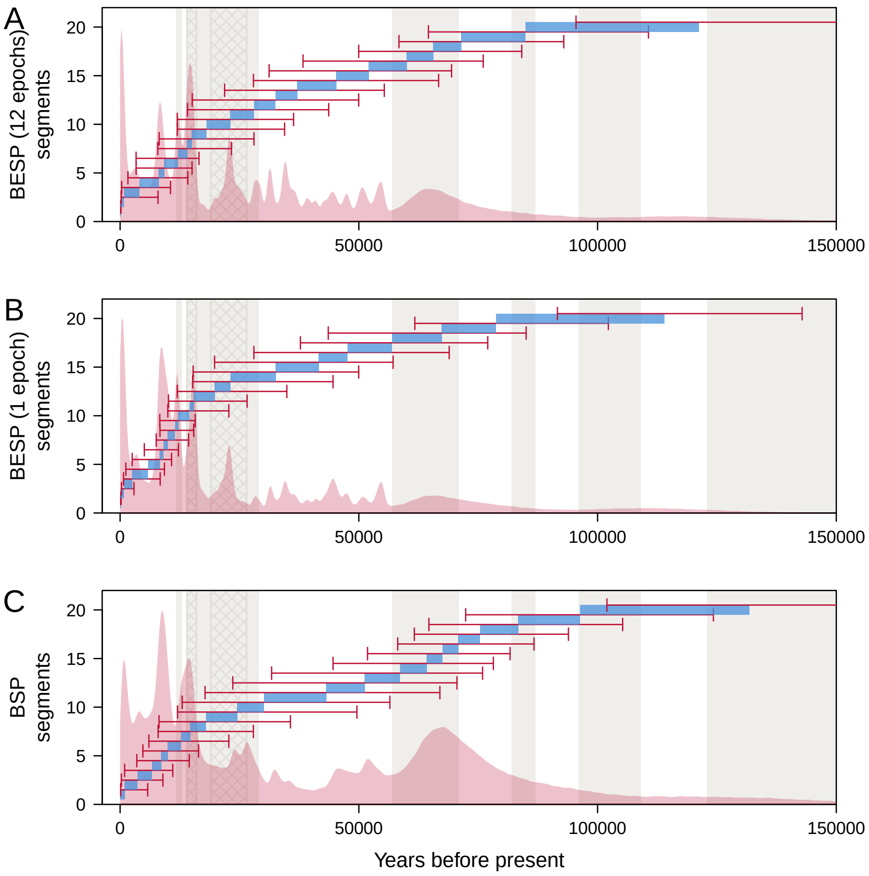

Figure S10: Population size segments for the alignment of 152 bison mtDNA sequences, as estimated under the 12-epoch BESP (A), single-epoch BESP (B) and BSP (C), with $p = 20$. Median posterior estimates of segments (t_{j-1}–t_j) are shown in blue. HPD intervals for the segment end-times are indicated by red arrows. Red shading shows the kernel density estimate of the posterior segment times (t_j). Grey shading indicates cool periods in the Earth's climate (from the present: Younger Dryas, Marine Isotope Stages (MIS) 2, MIS 4). The two cross hatched areas delimit the time of the last glacial maximum (≈26.5–19 ka BP) and approximate time of substantial human settlement of the Americas (≈16–14 ka BP).

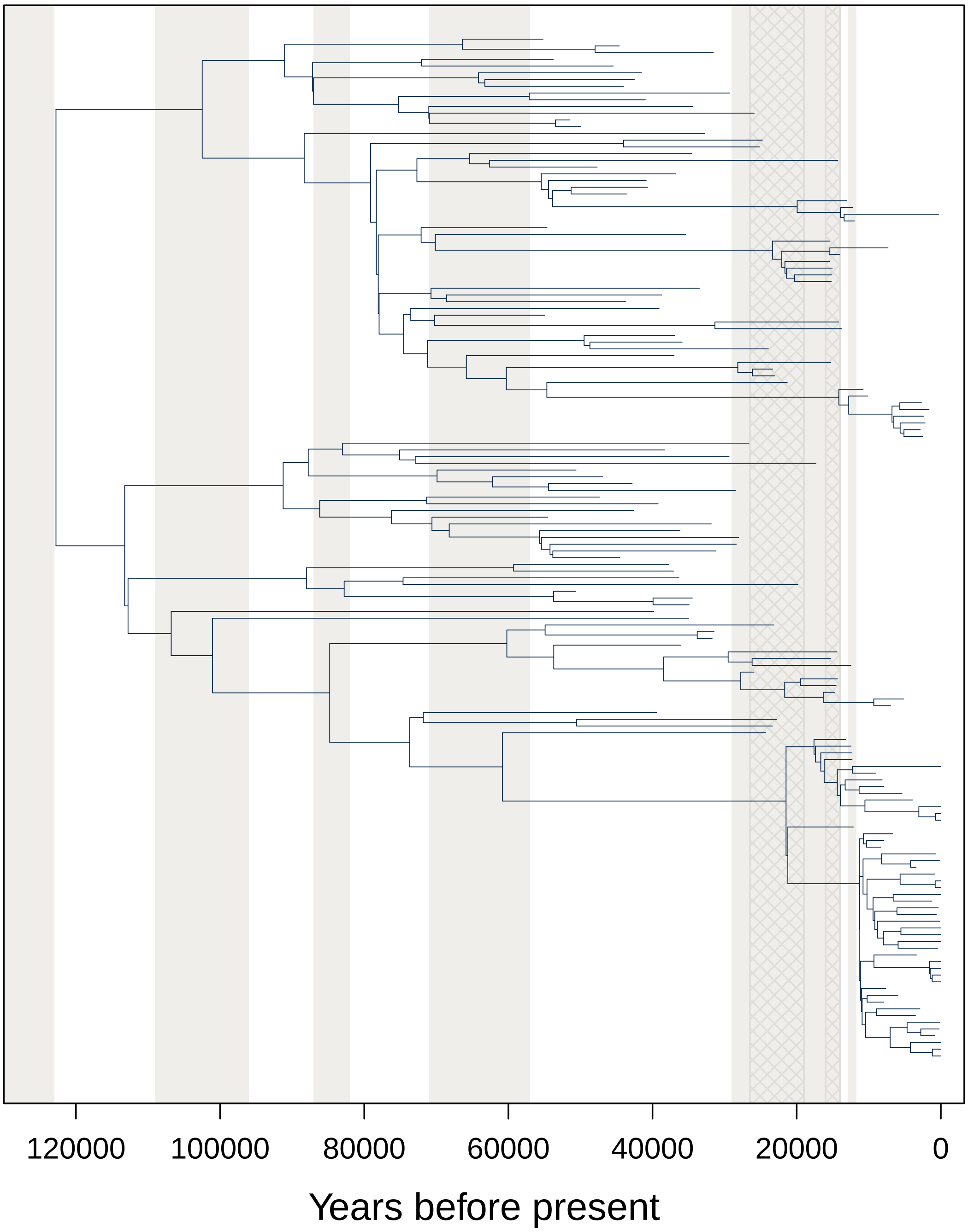

Figure S11: MCC tree of the alignment of 152 bison mtDNA sequences estimated under the 12-epoch BESP. Grey shading indicates cool periods in the Earth's climate (from the present: Younger Dryas, Marine Isotope Stages (MIS) 2, MIS 4). The two cross hatched areas delimit the time of the last glacial maximum (≈26.5–19 ka BP) and approximate time of substantial human settlement of the Americas (≈16–14 ka BP).
